# Supplementary material for: Transition of Carbon Nanotube Sheets from Hydrophobicity to Hydrophilicity by Facile Electrochemical Wetting
Source: Nanomaterials (Basel). 2023 Oct 26;13(21):2834. doi: 10.3390/nano13212834 (PMC10650619; doi:10.3390/nano13212834)
Supplement: Supplementary file 1 [file nanomaterials-13-02834-s001.zip › nanomaterials-2675104-supplementary.pdf]

Supplementary Information

Transition of Carbon Nanotube Sheets from Hydrophobicity to Hydrophilicity by Facile Electrochemical Wetting

Myoungeun Oh[†, 1, 2], Hyunji Seo[†, 1], Jimin Choi [1], Jun Ho Noh[1, 2], Juwan Kim [1, 2] Joonhyeon Jeon[* 2, 3] and Changsoon Choi[*, 1, 2]

[1]*Department of Energy and Materials Engineering, Dongguk University, 30 Pildong–ro, 1–gil, Jung–gu, Seoul 04620, Republic of Korea*

[2]*Department of Advanced Battery Convergence Engineering, Dongguk University, 30 Pildong–ro, 1–gil, Jung–gu, Seoul 04620, Republic of Korea*

[3]*Division of Electronics & Electronical Engineering, Dongguk University-Seoul, 30, Pildong–ro 1–gil, Jung–gu, Seoul, 04620, Republic of Korea*

[*] Correspondence: cschoi84@dongguk.edu

[†] These authors contributed equally to this work.

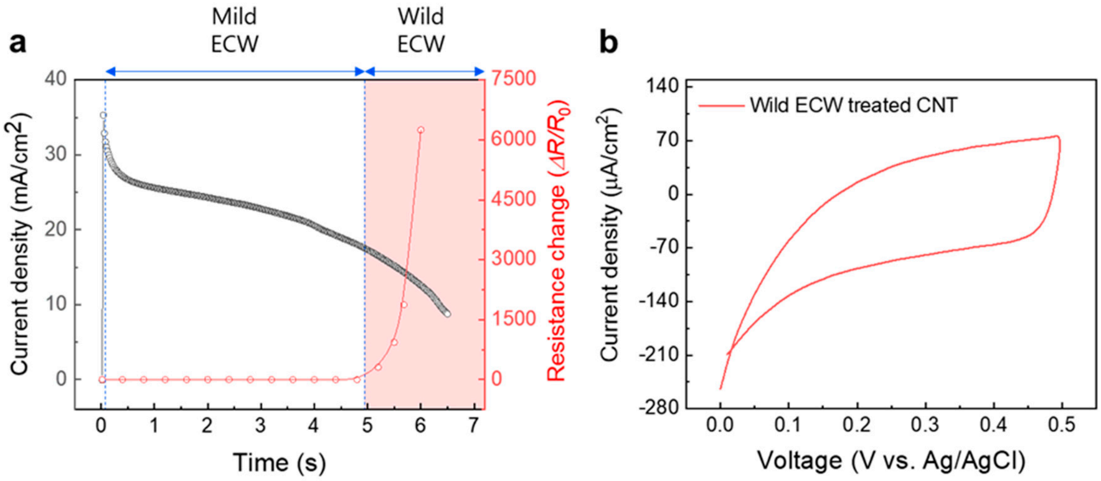

Figure S1. (a) Measured current density and resistance change of the CNT working electrode versus electrochemical wetting treatment time (the mild and wild electrochemical wetting regions are indicated). (b) CV curves of the wild–ECW–treated CNT sheet/PET film electrode.

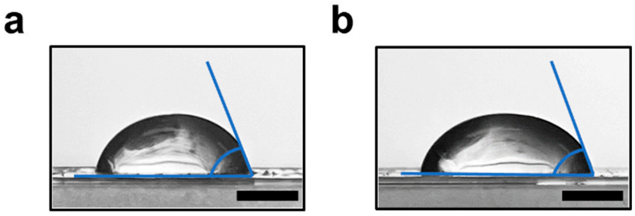

Figure S2. Actual images showing contact angles at applied voltages of (a) 2 V and (b) 4 V (scale bar = 0.25 cm).

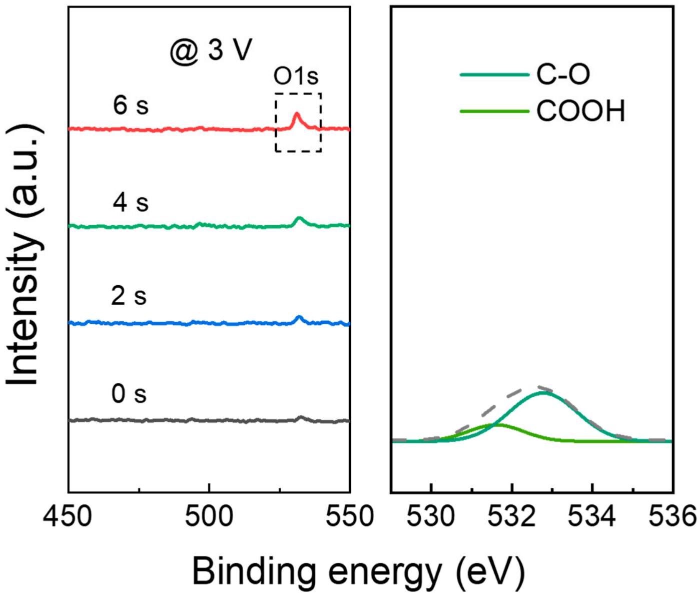

Figure S3. XPS survey spectra (left panel) and high-resolution O 1s XPS analysis spectra (right panel) of CNT sheets for various oxidation times at applied voltages of 3 V.

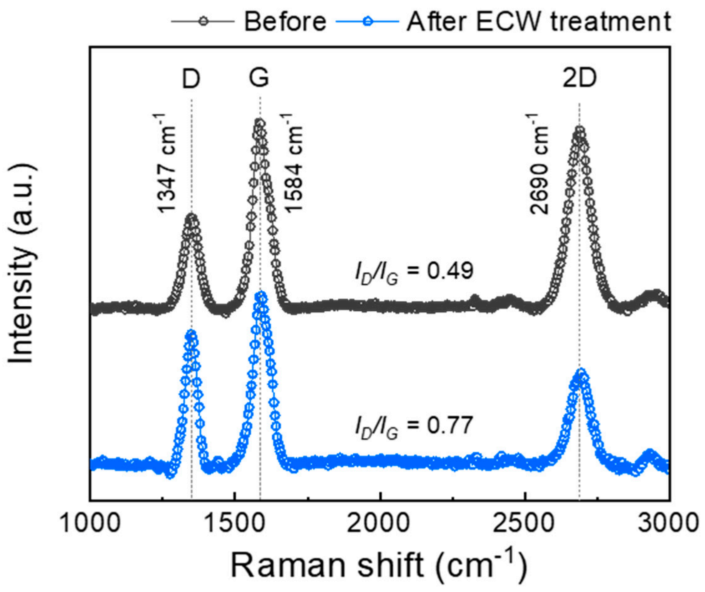

Figure S4. Raman spectra of the CNT sheets before (gray circle) and after ECW treatment (blue circle).
